# Neuronal signatures of network transition into bursting

**DOI:** 10.1186/1471-2202-14-S1-P432

**Published:** 2013-07-08

**Authors:** Sima Mofakham, Michal Zochowski

**Affiliations:** 1 Department of Biophysics, University of Michigan, Ann Arbor, Michigan 48009, USA; 2 Department of Physics, University of Michigan, Ann Arbor, Michigan 48009, USA

## 

The dynamics of neuronal networks in the brain can undergo sudden changes during an epileptic seizure. We are interested in identifying and characterizing the dynamical correlates of transition from asynchronous into synchronous network dynamics in order to detect the signatures of such a transition on the level of activity patterns of individual neurons. To do so, we investigated networks of excitatory and mixed excitatory-inhibitory which were composed of integrate-and-fire neurons in a one-dimension ring structure. We studied its dynamics as a function of the underlying structure of network connectivity, synaptic efficacy, level of external noise, and variability of natural spiking frequency. We investigated various correlates of network and neuronal dynamics just before bursting onset. Specifically, we looked at the evolving distributions of relative timing of neurons spikes right before the bursting and correlated them with the bursting onset. As a result of this correlation, we can identify the beginning of changes in the neuronal dynamics that lead to bursting transition, and measure lead-time within which we can predict transition into bursting based on the neuronal activity patterns. We showed that this lead-time is strongly influenced by network structure, noise level, and neuronal variability. We also demonstrated how this transition is influenced by inhibitory feedback and reduces the lead-time to transition.

